# Development and Validation of Attitude toward Gestational Surrogacy Scale in Iranian Infertile Couples

**DOI:** 10.22074/ijfs.2016.4776

**Published:** 2016-04-05

**Authors:** Fatemeh Rahimi Kian, Afsaneh Zandi, Reza Omani Samani, Saman Maroufizadeh, Abbas Mehran

**Affiliations:** 1 Faculty Member of Nursing and Midwifery Care Research Center, Tehran University of Medical Sciences, Tehran, Iran; 2 Department of Epidemiology and Reproductive Health, Reproductive Epidemiology Research Center, Royan Institute for Reproductive Biomedicine, ACECR, Tehran, Iran

**Keywords:** Surrogate Mother, Gestational, Attitude, Scale, Validation

## Abstract

**Background:**

Surrogacy is one of the most challenging infertility treatments engaging ethical, psychological and social issues. Attitudes survey plays an important role to disclosure variant aspects of surrogacy, to help meeting legislative gaps and ambiguities, and to convert controversial dimensions surrounding surrogacy to a normative concept that eliminates stigma. The aim of this study is to develop a comprehensive scale for gestational surrogacy attitudes.

**Materials and Methods:**

Development process of gestational surrogacy attitudes scale (GSAS) performed based on a descriptive cross-sectional study and included a rich data pool gathered from literature reviews, a qualitative pilot study on 15 infertile couples (n=30), use of expert advisory panel (EAP) consisting of 20 members, as well as use of content validity through qualitative and quantitative study by the means of content validity ratio (CVR) and content validity index (CVI). Also internal consistence using Cronbach’s alpha and test-retest reliability using intracalss correlation coefficient (ICC) were evaluated. Application of GSAS was tested in a cross-sectional study that was conducted on 200 infertile couples (n=400) at Royan Institute, Tehran, Iran, during 2014.

**Results:**

Final version of GSAS had 30 items within five subscales including "acceptance of surrogacy", "Surrogacy and public attitudes", "Child born through surrogacy", "Surrogate mother", and "Intentional attitude and surrogacy future attempt". Content validity was represented with values of CVR=0.73 and CVI =0.98. Cronbach’s alpha value was 0.91 for the overall scale, while ICC value due to test-retest responses was 0.89.

**Conclusion:**

Acceptable level of competency and capability of GSAS is significantly indicated; therefore, it seems to be an appropriate tool for the evaluation of gestational surrogacy attitudes in Iranian infertile couples.

## Introduction

Having a child is a universal desire (1) and considered mostly as a basis of human motivation for the continuity and stability of marriage (2). Infertility is defined as absence of pregnancy after one year of regular unprotected intercourse (3). Almost 10 to 15% of couples experience infertility (4). Infertile couples face pervasive personal and social crisis such as depression, anxiety, dissatisfaction and low selfesteem. Furthermore destructive impacts of infertility have been seen on interpersonal relationships, quality of life and marital status that may eventually lead to divorce (5). Due to importance of having children in Iranian culture, the social consequences of infertility goes deeper in conflicts (6).

In recent decades, there has been enormous improvement in infertility treatments. Surrogacy is one of the most challenging infertility treatment for which there has been considered lots of positive and negative outcomes (1), and includes two types of traditional and gestational surrogacy (7), although gestational type is the only type approved in Iran (8).

Choice of treatment for infertile couples is affected by their knowledge, understanding, expectations, as well as attitudes of the community they are living in; therefor, providing accurate information may have positive impact on a couple's decision-making (9, 10). Despite of consideration of surrogacy as legitimate treatment method, there is inadequate awareness in this respect in Iran (7, 11). People use attitudes to evaluate the objects by placing it within their existing knowledge structures, so attitudes influence the way they behave. It means that attitudes help an individual to decide whether behavior is appropriate or applicable. It is important to know about attitudes in order for predicting potential future behaviors (12).

Survey of knowledge, attitudes and decisionmaking patterns plays an important role in finding legal solutions in the process of converting surrogacy controversial approaches to a normative concept (11, 13). Nowadays, there is an increasing focus within the area of social psychology on developing methodologies that measure attitudes with self-report scales, while applications of these measures is required to continue development of methodologically strong and valid attitudes scales (12, 13). For ethical issues related to health-care, it seems to be essential to increase adequate awareness and to provide proper consultative services for infertile people (14). Furthermore disclosure of different aspects of surrogacy makes the relevant authorities to establish and to amend proportional regulations, leading to eliminate stigma in the community (9).

A few studies has been conducted in Iran in order to survey public opinion on gestational surrogacy (7). Scales used in the mentioned-studies are selfdesigned questionnaires with an indefiniteness in the expression of methods used in development for assessing validity and reliability of the scale. Therefore, we decided to conduct this study with the aim of achieving a valid gestational surrogacy attitudes scale which fulfilled all three cognitive, emotional and intentional aspects of attitude and conformed to cultural and logical aspects of surrogacy in Iranian infertile couples. This might be an opportunity to provide an organized and comprehensive scale that might best match their needs.

## Materials and Methods

Development process of gestational surrogacy attitudes scale (GSAS) was performed based on a descriptive cross-sectional study at Royan Institute, Tehran, Iran, during 2014. GSAS in Persian language was passed through the following steps:

### Development of a data pool

Initially literature review was conducted to the aim of development of concepts and questionnaire items, benefiting from related studies and an expert advisory panel (EAP) consisting of 20 members.

### Pilot study

A pilot study was performed on 15 infertile couples (n=30) who attended the Infertility Clinic of Royan Institute, with a previous knowledge of surrogacy issue. They were asked two open-ended questions including "What is your opinion about gestational surrogacy?" and "What is your most concerning issues about surrogacy experience?". Data pool was enriched with their answers as well as the comments provided by the members of EAP.

### Expert advisory panel

A team of experts in maternity and infertility issues consisted of 20 members as follows: 10 Academic Board Members of Nursing and Midwifery School, Tehran University of Medical Sciences, Tehran, Iran, and 10 specialists who were closely dealing with infertile couples and working at the different parts of the Royan Institute such as Academic Departments, Consultants and Treatment Clinics. Subsequently a primary version of the scale with 30 items retrieved from literature reviews and pilot qualitative study was judged and commented by the members of EAP. The final version of scale was then obtained after applying modifications for passing reliability and validity tests.

### Validity

Validation of the scale was performed in two ways of face validity and content validity. In face validity, difficulty, irrelevancy and ambiguity of scale items were assessed by the experts, in which they scored and qualified the scale according to the mentioned criteria and their recommendations. Content validity was carried out by both qualitative and quantitative approaches. Experts discussed and qualified the scale based on qualitative criteria including grammar, wording, item-allocation and scaling. Quantitative content validity was determined by the content validity ratio (CVR) and content validity index (CVI). For calculating CVR, each item of GSAS was scored by the members of EAP using a 3-point Likert scale as follows: i. Essential, ii. Useful but not essential, and iii. Unessential. According to Lawshe’s table, when there are 20 members in EAP, selected items are those with CVR= 0.42 or above (15).

According to Lawshe’s recommendation for determination of the mean values by the experts, which is assigned to each item of the scale, answers to CVR assessment questionnaire must be scored as 2 for "essential", 1 for "useful but nonessential", and zero for "unessential". Items with mean values more than 1.5 are acceptable, even though with CVR lower than 0.42 (15).

According to Lawshe’s recommendation for determination of the mean values by the experts, which is assigned to each item of the scale, answers to CVR assessment questionnaire must be scored as 2 for "essential", 1 for "useful but nonessential", and zero for "unessential". Items with mean values more than 1.5 are acceptable, even though with CVR lower than 0.42 (15).

### Reliability

Cronbach’s alpha and test-retest were used for evaluating the reliability of the scale (12). In internal consistence measured by Cronbach’s alpha, score equal to 0.7 or higher was considered as the acceptable reliability (17). Also stability of the questionnaire measured by test-retest method was determined. In order to define the coefficient for scale retesting, 15 infertile couples were randomly selected to answer the questionnaire twice within two weeks interval. Intracalss correlation coefficient (ICC) values equal to 0.4 or higher were considered acceptable (18).

### Descriptive study

Eventually at the end of the development process, with the aim of assessing practicability of GSAS, a descriptive cross sectional study on 400 infertile men and women were performed by simple sampling method with ethical considerations. Infertile couples participated in both pilot and final descriptive study with signing informed consents. This study approved by Tehran University of Medical Sciences, Faculty of Nursing and Miwifery and Reproductive Epidemiology Research Center of Royan Institute Ethics Committee.

## Results

Final version of GSAS was obtained with 30 items and each item was scored using five-point Likert scale; therefore, positive attitude expressions were scored as follows: I strongly agree=5, I agree=4, I am indecisive= 3, I disagree=2, and I strongly disagree=1. Negative attitude expressions were scored in the reverse order of the above-mentioned scoring, and these 14 items with negative connotations included item numbers of 6 to 11, 14, 15, 17, 18, 22 to 24 and 26.

Surrogacy is a concept associated with the most challenging issues that each aspect could lead someone to various and countercurrent attitude trends which could not to be totalized, thus according to the literature reviews and opinions of EAP, GSAS splits into five subscales including: i. Item numbers 1 to 9 indicating acceptance of surrogacy, ii. Item numbers 10 to 13 indicating surrogacy and public attitudes, iii. Item numbers 14 to19 indicating child born through surrogacy, iv. Item numbers 20 to 26 indicating surrogate mother, and v. Item numbers 27 to 30 indicating intentional attitude and surrogacy future attempt. Therefore, these items may cover major issues related to gestational surrogacy and prevent interference of opposite tendencies in attitude assessment.

Maximum score of the scale is 150, indicating more positive attitudes, while minimum score is 30. Score range defers in the subscales due to number of questions as it consists of 9-45 for overall acceptance of surrogacy subscale, 4-20 for surrogacy and public attitudes and intentional attitude and surrogacy future attempt subscales, 6-30 for child born through surrogacy subscale, as well as 7-35 for surrogate mother subscale. Both the total and subscale scores were calculated using raw scores after the negative items are recoded.

**Table 1:** Mean values of CVR, EAP, as well as face validity consisting of difficulty, irrelevancy and ambiguity in case of validation of GSAS items


Item number	Items	CVR	EAP mean value of judjment	Difficulty	Irrelevancy	Ambiguity

1	Surrogacy is a good way to help infertile couples have a child with their own genetic	80	1.9	100	100	100
	characteristics					
2	Surrogacy reduces psychological tensions in infertile couples	60	1.8	95	100	95
3	Surrogacy improves the life satisfaction of infertile couples	80	1.8	97.5	100	100
4	Surrogacy can prevent divorce and strengthen family structure	80	1.9	100	100	100
5	If there is no other infertility treatment op- tion, surrogacy could be the last choice	80	1.9	95	100	95
6	I prefer to be voluntarily childless rather than to accept surrogacy	80	1.9	100	100	97.5
7	Adoption is better than surrogacy	80	1.9	100	100	97.5
8	Surrogacy could be followed by ethical and.social issues	60	1.7	92.5	100	92.5
9	Surrogacy is against religion	80	1.9	95	100	97.5
10	Mainly most traditional societies have negative attitudes toward surrogacy	80	1.8	95	95	95
11	Surrogacy must be hidden from others inorder to prevent society to reject the child	100	1.9	90	92.5	92.5
12	I am not concerned about disclosure of sur- rogacy to friends and relatives	100	2	92.5	92.5	90
13	If mass media promotes public awareness about surrogacy, I will not be concerned about disclosure of the issue to the child and the others	80	1.9	95	95	92.5
14	Children born through surrogacy may have further risk of birth defects than others	60	1.9	97.5	100	97.5
15	Children born through surrogacy may have further risk of psychological problems than others	80	2	100	100	100
16	Disclosure of surrogacy is considered as an inalienable right of the child	80	2	100	100	100
17	Surrogate mother’s identity must be hidden from the child	60	1.9	100	100	100
18	Close relationship of the child and surrogate mother will cause insecurity of parental relationship between commissioning couple and the child	60	1.7	95	92.5	95
19	Disclosure of surrogacy to the child is better to be after his/her adolescence stage	80	1.9	100	100	100
20	Only commissioning couple are truly parents of the child	80	2	100	100	100
21	Surrogate mother’s role is as antenatal nanny	60	1.7	100	100	100
22	It seems that surrogate mother’s intention is to get money rather than to be altruistic	80	1.8	100	100	100
23	Surrogate mother might be careless about the child during pregnancy	80	1.9	100	95	100
24	Emotional bonding may cause surrogate mother to avoid relinquishment of the child	60	1.9	100	95	100
25	I prefer involving an unfamiliar surrogate mother	100	2	100	100	100
26	There is no need to maintain contact with surrogate mother after delivery	100	2	100	100	100
27	If my physician recommends to get a surrogate, I will use this treatment	100	2	100	100	100
28	If I know that one of my relatives or friends decide to be a surrogate mother, I will support them	100	2	100	100	100
29	In case of use of surrogacy, I will disclose the truth to my child in future	60	1.7	100	100	100
30	After relinquishment of the baby, facing the surrogate mother never make me uncomfortable	80	1.7	100	10	100
	Total scale	73	1.8	98	98	98


CVR; Content validity ratio and EAP; Expert advisory panel.

**Table 2:** ICC and Cronbach’s alpha values of subscales


Subscales	ICC	Cronbach’s alpha

Overall acceptance of surrogacy	0.90	0.93
Surrogacy and public attitudes	0.86	0.90
Child born through surrogacy	0.73	0.91
Surrogate mother	0.87	0.91
Intentional attitudes and surrogacy future	0.82	0.93
Total scale	0.89	0.91
ICC; Intracalss correlation coefficient.		


ICC; Intracalss correlation coefficient.

In this study, total values of CVR and CVI were 0.73 and 0.98, respectively, representing the substantial content validity of the scale. Mean value of the judgment status of expert panel was calculated above 1.5 for each item; therefore, they were all accepted in the final version of the scale. Reliability of GSAS has confirmed by Cronbach’s alpha value that was 0.91 for the overall scale, and the value of ICC due to test-retest responses was found 0.89 (Tables1, 2).

Application of GSAS was tested in a cross-sectional study on 200 infertile couples who were applied to receive infertility treatment service at Royan Institute (n=400) via simple sampling method. The mean age of men was 34 ± 5.52 and for women was 29 ± 5.12. They were all Muslim, among which 97.5% were Shiite. Among 200 infertile couples, 55 women (27.5%) and 49 men (24.5%) showed an education level of elementary school, 72 women (36%) and 65 men (32.5%) had a high-school diploma, as well as 73 women (36.5%) and 86 men (43%) had university degrees. Mean of infertility duration was 4.12 ± 2.74 years. Mean score of total attitude toward gestational surrogacy was 91/14 in women and 92/46 in men, indicating there was no significant difference between couples. Results of this study proved the practicability of the scale.

## Discussion

Due to the importance of surrogacy issue, similar surrogacy attitudes surveys have been conducted around the world and the majority of them were used self-designed questionnaires in accordance with the objectives of their studies, but the validation methods are not clearly represented. One of the privileges of GSAS compared to the tools used in those studies is the application of an enriched scientific and statically validation process.

Here we pointed the most cited surrogacy attitudes- related articles to compare scale development approaches. Members of EAP also provided more erudite comments for developing a satisfactory scale in practice as compared to other sources used in this study. As results, Cronbach’s alpha value indicates an excellent reliability of the scale in this study that is similar to the studies by Ahmari Tehran et al. (19), Rahmani et al. (20). However, in the studies by Saito and Matsuo (21) as well as Poote and van den Akker (22), Cronbach’s alpha or other validity and reliability assessments was not applied. In order for item-designing and -scoring, Saito and Matsuo (21) and Minai et al. (23) used open ended questions, whereas present study and other studies used Likert scale (mostly 5-point). Furthermore, scale development in Poote and van den Akkerʼs (22) study was based on theory of planned behavior (TPB). In the present study, 30 different pilot studies were employed that is similar to the study by Chliaoutakis et al. (24), whereas other studies, either included no pilot study, or had less pilot sample size. This study seems to be unique among previous surrogacy attitudes surveys. Firstly we performed qualitative pilot study in order to enrich data pool, and we also applied the qualitative and quantitative content validity approaches to obtain highly acceptable CVR and CVI values. Finally we attained the test-retest ICC values within satisfactory level.

Content validity is an essential step in the development of new empirical measuring devices because it represents a beginning mechanism for linking abstract concepts with observable and measurable indicators (25); however, none of the mentioned studies reported CVR and CVI values. As a result, studies based on only relevance or representativeness, as judged by experts, cannot offer any support for validity. Therefore, it is important to abandon content validity, to clarify subscales, and to develop the adequacy of content sampling from the content domain for GSAS (26).

In this study, we tried to take into account these most important criteria and other prestigious methods for development and validation of the scale. Therefore, after obtaining satisfactory levels in validity and reliability process, our findings significantly indicated the adequate level of competency and capability of applied scale in disclosure of tendencies toward major challenging aspects of GSAS.

## Conclusion

According to the results, GSAS provides enough admissibility and validity in evaluation of gestational surrogacy attitude in Iranian infertile couples, so seems to be useful in surveys with similar sociocultural backgrounds. Further studies within different populations are suggested to determine if the scale can accurately identify attitude toward gestational surrogacy in variable demographic characteristics and cultural backgrounds.
